# Multi-object detection for crowded road scene based on ML-AFP of YOLOv5

**DOI:** 10.1038/s41598-023-43458-3

**Published:** 2023-10-12

**Authors:** Yiming Li, Kaiwen Wu, Wenshuo Kang, Yuhui Zhou, Fan Di

**Affiliations:** 1 College of Electronic and Information Engineering, Shandong University of Science and Technology, Qingdao, 266590 China; 2 Hunan University, Changsha, 410082 China; 3 National Engineering Research Center of RVC, Changsha, 410082 China

**Keywords:** Electrical and electronic engineering, Information technology

## Abstract

Aiming at the problem of multi-object detection such as target occlusion and tiny targets in road scenes, this paper proposes an improved YOLOv5 multi-object detection model based on ML-AFP (multi-level aggregation feature perception) mechanism. Since tiny targets such as non-motor vehicle and pedestrians are not easily detected, this paper adds a micro target detection layer and a double head mechanism to improve the detection ability of tiny targets. Varifocal loss is used to achieve a more accurate ranking in the process of non-maximum suppression to solve the problem of target occlusion, and this paper also proposes a ML-AFP mechanism. The adaptive fusion of spatial feature information at different scales improves the expression ability of network model features, and improves the detection accuracy of the model as a whole. Our experimental results on multiple challenging datasets such as KITTI, BDD100K, and show that the accuracy, recall rate and mAP value of the proposed model are greatly improved, which solves the problem of multi-object detection in crowded road scenes.

## Introduction

Object detection in road scene is one of the core problems of intelligent traffic monitoring, which is mainly divided into detection model based on background network and feature network. However, due to the complex road scene, diverse weather conditions, vehicle occlusion and light change, the existing target detection effect is not ideal^1^.

At present, the object detection model based on deep learning are mainly divided into two categories, one is Anchor-free model, and the other is Anchor-based model. In Anchor-free model, each sample point is directly used as the starting point for prediction by backbone. As long as the position falls into the ground truth, the point is considered as a positive sample and trained. This approach avoids the problem of unbalanced sample distribution caused by too many anchors, reduces the calculated amount and improves the efficiency. Its representative network is FCOS^2^, CenterNet^3^, CornerNet^4^. The Anchor-based model means that in the target detection task, some anchor boxes of inherent size and shape are set in advance to predict the position and size of the target, so that the network can directly classify the object and regression the bounding box on the basis of the box, and its training is stable. In the meantime, dense anchor boxes can effectively improve the recall ability of the network for the target. The representative network is Faster-RCNN^5^, YOLOv3^6^.

Although the overall object detection model has made great progress, there is still a big improvement space for optimization in the performance of special scenes, such as cross occlusion, motion blur, and small objects in the image, which lead to poor detection results. References^7–9^ take into account the problem of difficult small target but do not consider the occlusion problem in complex scenes. Documents^10–13^ instead consider the occlusion problem but do not consider the difficulty of small target detection.

For the occlusion problem, Wang et al.^14^ proposed the Repulsion Loss, which makes the candidate bounding box closer to the specified target while moving away from the background. In order to prevent the prediction box from moving to adjacent targets and overlapping effectively. Zhang et al.^15^ presented Occlusion-aware R-CNN, which improves the ROI Pooling layer and uses the element-wise sum to merge the features of all sub-regions for the final classification and regression. Chu et al.^16^ proposed simple and effective multi-instance prediction, EMD loss method and Set NMS and an optional refinement module (RM) to supervise the learning of instance set prediction and suppress the repetition in different schemes. The complexity of the network also increases after adding multiple methods. Wanchaitanawong et al.^17^ proposed a multi-modal RPN with a regressor and classifier for each mode to adjust the bounding box position and confidence scores and introduced a new evaluation metric, "multi-modal IoU (IoUM)". The detection effect is not optimal when the target is misaligned. Hou et al.^18^ proposed regional feature completion (RFC), which designed SRFC and TRFC modules to capture spatial and temporal background to restore closed areas, although this design prefers static scene features. For the problem of small-target detection, In 2021, Lim et al.^19^ proposed an improved network of FA-SSD for small object detection, which introduced feature fusion to obtain context information, and added a module with attention to enable the network to focus on important parts, which greatly improved the detection accuracy of small objects. However, after the introduction of the above modules, the number of network layers becomes more, the processing time increases and the extraction of the small target features is not sufficient. Deng^20^ found although different scales of feature fusion can improve the detection ability of small targets, but in the process of feature mapping size the target will share features, so proposed an extended feature pyramid network, establish high resolution features map for small target detection, however, the network cannot take into account the detection effect of large, medium and small goals. Liu et al.^21^ proposed a high-resolution detection network, which uses a shallow network to process high-resolution images and a deep network to process low-resolution images. It extracts more semantic information while retaining as much as possible location information of small objects, and improves the detection performance of small objects. The detection performance of the network is determined by the resolution, and the mAP value can reach 85.7%.

At present, some progress has been made in the detection of small targets, but such challenging problems in crowded road scenarios are far from being solved, such as less small target feature extraction, multi-target cross-occlusion, and insufficient model feature extraction. Our contributions are as follows: In view of the above multi-objective detection problem in crowded road scenarios, the paper proposes a multi-objective detection model of YOLOv5 based on the improved ML-AFP mechanism, which mainly solves the detection problem of motor vehicles, non-motor vehicles and pedestrians on the road. The model achieves good results in different scenarios and shows great improvements in precision and recall.

The innovation of this article includes two points:

1. For the neck part of YOLOv5, the ML-AFP mechanism is proposed to enable the adaptive fusion of spatial feature information at different scales to improve the distinguishing ability of local regional convolution and the detection ability of dense repeated samples.
2. In view of the problem of difficult small target detection, the paper proposes to add the small detection layer in YOLOv5 network structure to improve the detection ability of small targets; In the head part of network structure, double detection header is used to predict the classification and regression information to improve the overall detection ability of the model. On the problem where dense object detection is difficult, the model uses varifocal loss's classification loss function to produce a more accurate ranking in the dense object detector.

In the second section, the overall framework of the model is introduced, and the network model is improved for the detection of crowded road scenes. Each improvement point is explained. The third section introduces the ML-AFP mechanism proposed in this paper. The fourth section shows the experimental part of this paper, and analyzes the experimental results.

## The overall framework and the improvement of the multi-class object detection model

### Overall framework of the model

**Figure 1:**
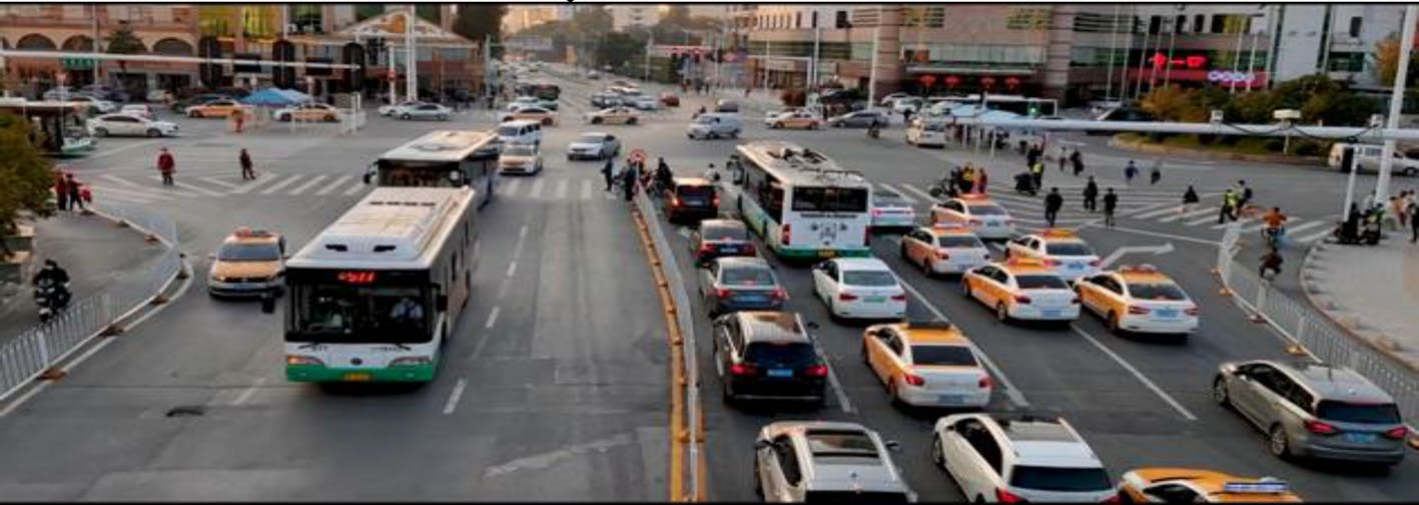
Real road scene.

Object detection model based on deep learning rely on powerful feature extraction capabilities to avoid the influence of illumination, background and other factors on detection results. However, there are still some difficulties and challenges for different scenes. In the real road scene, vehicles, non-motor vehicles and pedestrian emerge in an endless stream, and sometimes they are too dense and crowded, as shown in Fig. 1.

The YOLOv5 model inherits the anchor base mechanism of the previous generation of YOLO model. What is different is that the innovative YOLOv5 model is embedded with the adaptive anchor box mechanism. The previous model need to use the model alone to calculate the anchor frame value, while the YOLOv5 embedded anchor base value computer system greatly improves the efficiency of the model. Images entering YOLOv5 first undergo a Mosaic data augmentation method. Mosaic uses random scaling, random cutting and random arrangement of images, which enriches the data set, improves the effect of small target detection, and enhances the robustness of the model. YOLOv5 The boundary box regression loss function of the output end adopts CIOU_Loss, which fully takes into account the three important geometric factors of overlap area, center point distance and aspect ratio, improves some occlusion overlapping targets, and greatly improves the speed and accuracy of prediction frame regression. YOLOv5 The model directly calls the Pytorch official NMS method (Non-Maximum Suppression), which mainly screens the candidate box through IoU (Intersection over Union), thus causing the following problems: First, when the road target is too close, NMS will directly delete the detection box beyond the set threshold, resulting in a decrease in detection accuracy. Second, NMS must manually set the threshold, can not well adapt the model. Therefore, it needs to be improved in the occlusion target and small target detection.

**Figure 2:**
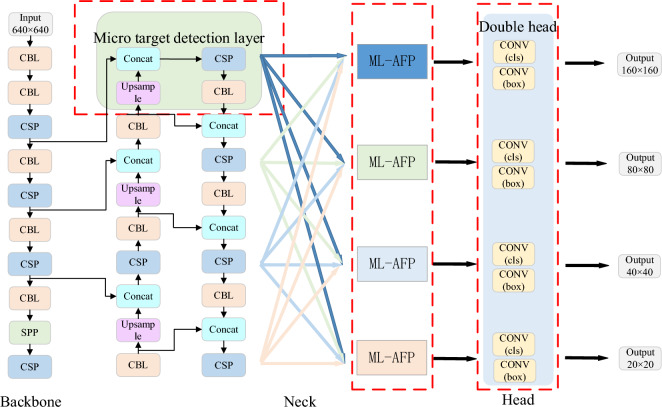
The improved YOLOv5 multi-class object detection network.

Therefore, we construct a multi-class object detection model based on YOLOv5 and ML-AFP mechanism, and its framework is shown in Fig. 2. Firstly, Backbone features are extracted from the input image, and the features of different scales are obtained by convolutional downsampling. A small object detection layer is added to focus on extracting small object information for subsequent detection. Then, the extracted features are passed through FPN (Feature Pyramid Network)^22^ and PANet^23^ realizes feature fusion of up-sampling and down-sampling. The fused features are spatially adaptively refined by the designed ML-AFP mechanism, so that the network can pay more attention to useful information. Finally, the double head mechanism is used to extract the classification information and regression information respectively, and the non-interference between classification and regression makes the detection accuracy higher.

### Improvement for small object detection

#### Improvement of detection layer

In the process of extracting features from the backbone, YOLOv5 network goes through three downsampling, which are Level 3, Level 2, and Level 1. Small objects occupy a small proportion in the original image, with the downsampling process, small objects only account for a single digit pixel size in the feature map. Considering that the feature area of the tiny target is too small, the characteristics of the target may disappear before passing Level 3. Therefore, in Fig. 2, the detection layer Level 4 of the tiny target is added to the second downsampling of Backbone to pay attention to the learning process of small targets and help the whole detection network to better detect small targets. The added small target detection layer and the feature layers of the other three scales are fused at multiple scales through the feature pyramid structure FPN and PANet to enhance the full utilization of features. In the final detection stage, the detection layer complexly detects small targets.

#### Double head mechanism

In the Head prediction part of YOLOv5 network structure, 1 × 1 convolution is used to predict the classification task and regression task of the whole target. Due to the contradiction between the classification task and the regression task, using a 1 × 1 convolution does not separate the two tasks well. Therefore, in Fig. 2, the Head part of YOLOv5 is improved in this paper, and the double head mechanism is used to predict the classification information and regression information respectively. After the multi-scale fusion of the Neck part, the input feature vector of each prediction feature layer is $F_{c}^{in}$, where $c$ is the convolution channel number. In an input feature vector, different channels predict different information, therefore, according to the channel position $F_{c}^{in}$ will be divided: $F_{c}^{in} = \left[ {F_{c1}^{box} ,F_{c2}^{obj} ,F_{c3}^{cls} } \right]$, $F_{c1}^{box}$ represents the regression information of the detection box, $F_{c2}^{obj}$ represents the confidence information of the detection box, $F_{c3}^{cls}$ represents the classification information of the detection box, and $c = c1 + c2 + c3$. In this paper, 1 × 1 convolution is used to predict different information according to the location of the channel, and its predicted information is integrated in the channel dimension, as follows:

1 $$F_{c}^{out} = Concat(conv_{1 \times 1} (F_{c1}^{box} ) + conv_{1 \times 1} (F_{c2}^{obj} ) + conv_{1 \times 1} (F_{c3}^{cls} ))$$

### Improvement for occluded object detection

In the process of NMS, the detection box with the highest confidence is selected as the benchmark, and then the IOU between the detection box and other detection boxes in the same category is calculated. If the IOU exceeds the set threshold, it is removed. The above steps are repeated until all the detection boxes are processed. Although NMS can effectively filter out duplicate detection boxes, there are still some problems in the processing of cross-occlusion objects in dense scenes. Due to the small number of occluded target features, the confidence score of model prediction is low. When NMS uses classification confidence to sort, the detection boxes with low scores and high IOU but predicted pairs will be filtered out, resulting in low recall of the whole model. Therefore, this paper adopts Varifocal loss in terms of confidence loss and classification loss. This loss function can represent the location-aware of object presence and localization accuracy or the IOU-aware classification score loss at the same time, so as to produce more accurate ranking on dense object detectors. The Varifocal loss function is as follows.

2 $$VFLoss = \left\{ {\begin{array}{*{20}l} { - q(q\log ^{p} + (1 - q)\log ^{{(1 - p)}} )} \hfill & {q > 0} \hfill \\ { - \alpha p^{\gamma } \log ^{{(1 - p)}} } \hfill & {q = 0} \hfill \\ \end{array} } \right.$$

where,$p$ is the prediction score, $q$ is the target IOU score, $\alpha$ and $\gamma$ is the weight. This loss function can use positive samples to supervise the signal, so that the model can focus on high-quality samples during training.

## ML-AFP multi-level feature aggregation for multi-class object detection

Since the targets detected in this paper are vehicles, non-motor vehicles and pedestrian, we need the network to be more sensitive to the extracted features and feature fusion. Therefore, we propose ML-AFP mechanism to achieve highly accurate detection of multi-class objects. The ML-AFP mechanism makes full use of the spatial information collected by the pooling kernel and the cross-channel feature information extracted by the ordinary convolution kernel. Feature aggregation can make full use of the semantic information of high-level features and the fine-grained features of low-level features, integrate the information of different levels, and enhance the feature expression ability of the network. At the same time, the pooling module and 1 × 1 convolution are introduced to capture the feature response between spatial position and cross-channel. Finally, the Sigmoid function and residual connection are used to supplement the information to strengthen the long-distance dependence of features.

**Figure 3:**
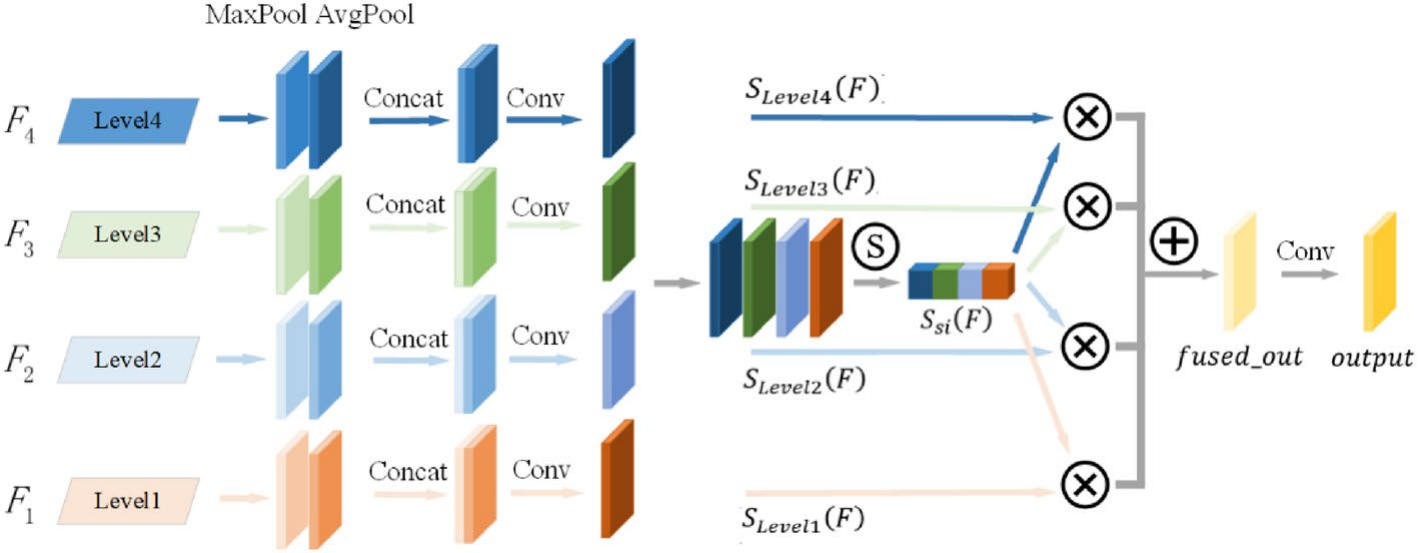
Multi-level aggregation features perception.

Figure 3 shows the mechanism of multi-level aggregation feature perception. Level 1-Level 4 uses FPN and PANet structures to output feature maps of different scales respectively. For each level, the feature maps of the other three different levels and different scales are firstly integrated into the same scale by up-sampling or down-sampling, and then four feature maps of the corresponding level and the same scale are formed $F_{i} (i = 1,2,3,4)$. The obtained feature maps are then aggregated by the maximum pooling module and the average pooling module, which can better fuse the channel information to extract features without changing the spatial dimension. In addition, the module uses different pooling methods at adjacent scales to improve the discernibility of convolution in local regions. Then, the concat method is used to concatenate the pooled feature maps to superpose the spatial features of the target. Finally, the integrated information is compressed by 1 × 1 convolution to capture the dependencies between channels, so that the mechanism has the ability to learn the interaction between each channel, output $S(F) = [S_{level1} (F),S_{level2} (F),S_{level3} (F),S_{level4} (F)]$. The specific calculation formula of this module is shown below.

3 $$S_{Leveli} (F) = Conv_{1 \times 1} (Concat([AvgPool(F_{i} );MaxPool(F_{i} )])) \, i = 1,2,3,4$$

After obtaining the integrated spatial information $S(F)$, the multi-level features of the four levels were fused in the channel dimension. Since the detection targets of this paper are vehicles, non-motor vehicles and pedestrians, multiple class labels need to be predicted, and the class labels are not mutually exclusive, the nonlinear activation of the Sigmoid function is used to calculate the weight of each Level layer. Considering that the convolution will compress the channels when obtaining the degree of dependence between channels, resulting in the loss of the spatial information of the feature map to a certain extent, the fusion weight is adaptively adjusted by multiplying with the corresponding level to learn the contribution of different scales to the prediction feature map. Finally, the 1 × 1 convolution output is used to combine the information, and the formula is as follows.

4 $$\left\{ \begin{gathered} [S_{s1} (F),S_{s2} (F),S_{s3} (F),S_{s4} (F)] = Sigmoid(Concat[S(F)]) \hfill \\ fused_{out} = \sum\nolimits_{i = 1}^{4} {S_{Leveli0} (F) \cdot S_{si} (F)} \hfill \\ output = Conv_{1 \times 1} (fused_{out} ) \hfill \\ \end{gathered} \right.$$

The ML-AFP mechanism proposed in this paper is plug-and-play, and the pooling module in the structure can integrate the spatial information of different types of targets. At the same time, the 1 × 1 convolution can model the important relationship between channels to output the global feature response better. The mechanism can also dynamically adjust the importance of different levels of feature maps, which can focus on learning the features of this layer and filter the features of other levels. At each spatial location, features of different levels are adaptively fused to retain useful information.

## Analysis of experimental results

### Experimental environment and design

The experiment of multi-class target detection are: vehicle, non-motor vehicle and pedestrian and their corresponding detection label are “car”, “bike”, and “person”. In this paper the detection framework is carried out in an environment named Anaconda, implemented based on Pytorch and Python, and GPU graphics card is used to accelerate the calculation.

Two datasets are used in this experiment: KITTI dataset jointly created by Karlsruhe Institute of Technology (Germany) and Toyota American Technical Research Institute (Toyota American Technical Research Institute)^24^ and the BDD100K dataset published by Berkeley AI Lab^25^. These two datasets have a large amount of data and various data types, which can better simulate real road scenes. There were 6500 images in the KITTI training set and 981 images in the validation set. The labeled targets in the dataset include about 29,000 vehicle targets, about 2500 non-motor vehicle targets, and about 5000 perosn targets. The BDD100K training set had 70,000 objects and the validation set had 10,000 objects. The labeled targets in the dataset include about 700,000 vehicle targets, about 15,000 non-motor vehicle targets, and about 100,000 pedestrians.

mAP (Mean Average Precision) is a very important evaluation index to measure the accuracy of the detection model. Its size is related to the Precision (P) and Recall (R) of the detection results. P refers to the proportion of correctly detected positive samples and all positive samples detected, and R refers to the proportion of correctly detected samples and all positive samples in the dataset. The P-R curve can well reflect the relationship between precision and recall. In general, the performance of the model can be measured by the size of the area enclosed by the P-R curve, the larger the area, the better the model.

In the training time, the input image size is 640 × 640, batchsize is 16, the number of training rounds is set to 100 for BDD100K, the number of training rounds is 60 for KITTI, the IOU threshold is 0.5, the initial learning rate is 0.01, the learning rate decay method is cosine annealing, and SGD optimization is used for gradient descent. The momentum parameter was set to 0.937, and the weight decay was 0.0005. The weight of the classification loss is 0.5, the weight of the confidence loss is 1.0, and the weight of the regression loss is 0.05.

In this paper, we conduct two parts of experiments for multi-class object detection model. The first part is the comparison experiment of the improved model with the two-stage detection model and the Anchor free detection model, and the ablation experiment of each improved measure. The other part mainly compares the ML-AFP mechanism proposed in this paper with other improved feature pyramid structures.

### Experiment and effect of detection model

**Figure 4:**
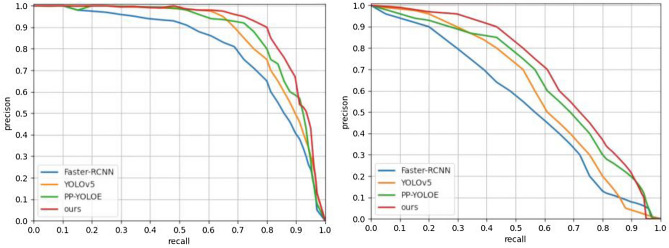
P-R curves.

This paper compares Faster-RCNN, YOLOv5, and PP-YOLOE^26^ The P-R curve of the model and the improved YOLOv5 model in this paper, and the experimental results are shown in Fig. 4. The left part of Fig. 4 is the P-R curve of the above model in the KITTI dataset, and the right part is the P-R curve of the above model in the BDD100K dataset. It can be seen from the Figureure that under the premise of IOU = 0.5, the area of the P–R curve enclosed by the improved model is the largest in this paper, indicating that the improved network model detection performance of this paper is better.

In addition, Faster-RCNN, YOLOv5, and PP-YOLOE model were evaluated by using AP of each category as indicators, and ablation experiments were carried out for each improved module of YOLOv5 in this paper. The experimental results are shown in Tables 1 and 2.

**Table 1:** Comparison of different model and ablation experiment on BDD100K.

Model	mAP (%)	Person (%)	Bike (%)	Car (%)
Faster-RCNN	50.7	49.4	34.2	68.6
YOLOv5	59.0	56.1	43.5	77.3
PP-YOLOE	59.8	58.1	46.1	75.2
YOLOv5(Varifocal loss)	56.7	54.2	40.8	75.1
YOLOv5(double head)	63.1	60.8	48.8	79.7
YOLOv5(double head + micro target detection layer)	65.7	64.2	50.1	82.9
YOLOv5(double head + micro target detection layer + VF loss + ML-AFP)	66.8	65.7	51.0	83.7

**Table 2:** Comparison of different model and ablation experiment on KITTI.

Model	mAP (%)	Person (%)	Bike (%)	Car (%)
Faster-RCNN	78.4	69.0	80.8	85.4
YOLOv5	88.4	78.4	90.4	96.2
PP-YOLOE	88.8	79.3	89.9	97.4
YOLOv5(Varifocal loss)	84.2	73.5	84.5	94.6
YOLOv5(double head)	89.8	80.7	91.6	97.0
YOLOv5(double head + micro target detection layer)	91.2	83.5	92.7	97.3
YOLOv5(double head + micro target detection layer + VF loss + ML-AFP)	**93.2**	**87.9**	**94.4**	**97.5**

Significant values are in bold.

**Figure 5:**
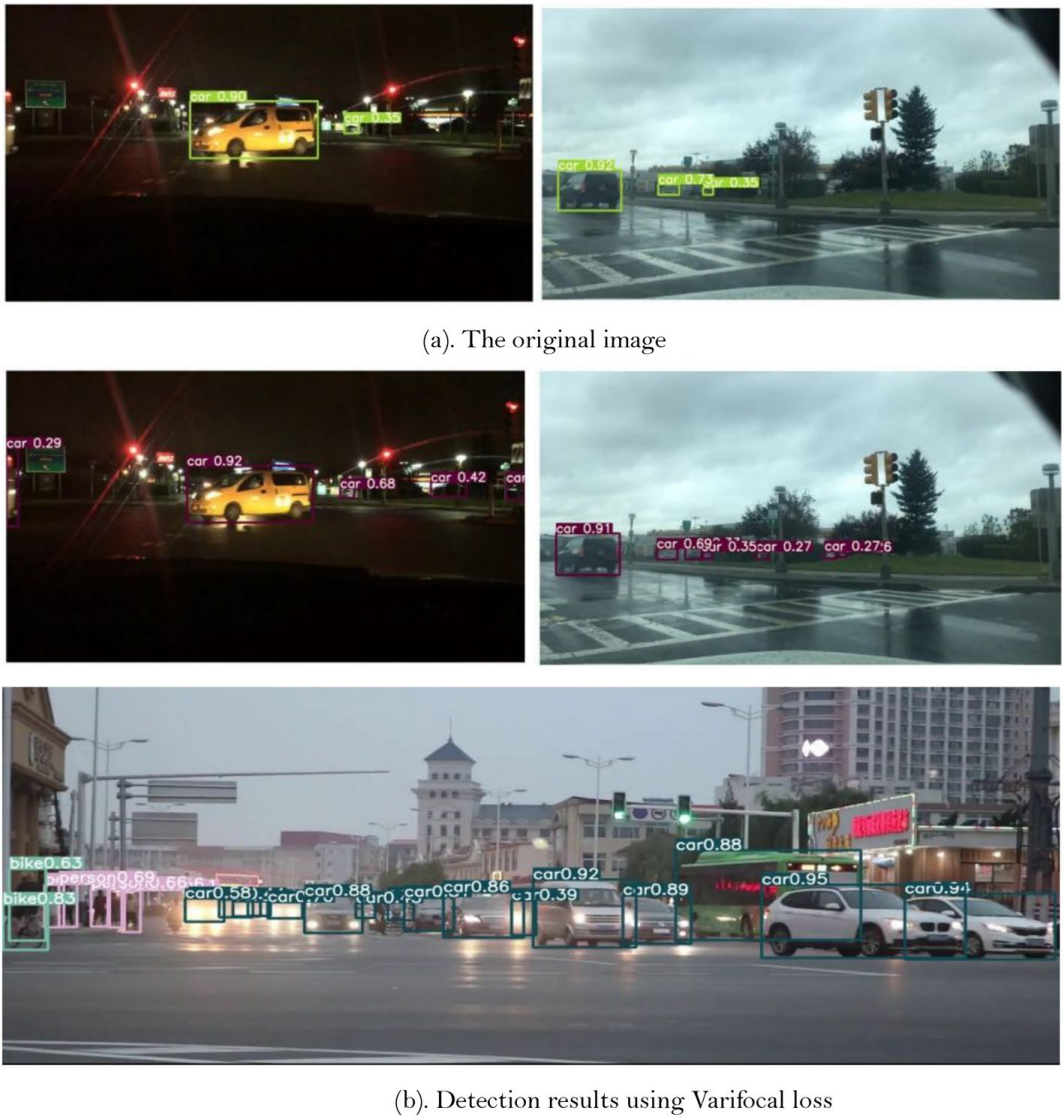
(**a**) The original image. (**b**) Detection results using Varifocal loss.

From the results in Tables 1 and 2, it can be seen that the mAP and AP of Fast-RCNN and YOLOv5 are relatively low compared with other model, which is difficult to meet the requirements of real-time detection. Although the PP-YOLOE detection model has some improvement in indicators, for dense targets and small targets existing in road scenes, it is difficult to achieve real-time detection and its detection effect is poor. In addition, in order to investigate the effect of the improved measures of ML-AFP, double head, tiny object detection layer, and Varifocal loss designed in this paper. In the meanwhile, it conducts ablation experiments on each improved module. It can be seen from Tables 1 and 2 that although the mAP value decreases somewhat after using Varifocal loss to calculate the loss of classification and confidence, it is 56.7% and 84.2% on KITTI dataset and BDD100K dataset, but due to the loss function improves the correlation between target classification score and positioning accuracy, reduces the impact of filtering the occluded targets with high IOU value but low score caused by only using classification confidence ranking in the NMS, and improves the recall of the detection model to a certain extent. Therefore, the recall of the overall detection model is improved at a certain loss of accuracy, so that more targets are detected, which can be used for the detection of real road scenes. The detection effect is shown in Fig. 5.

**Figure 6:**
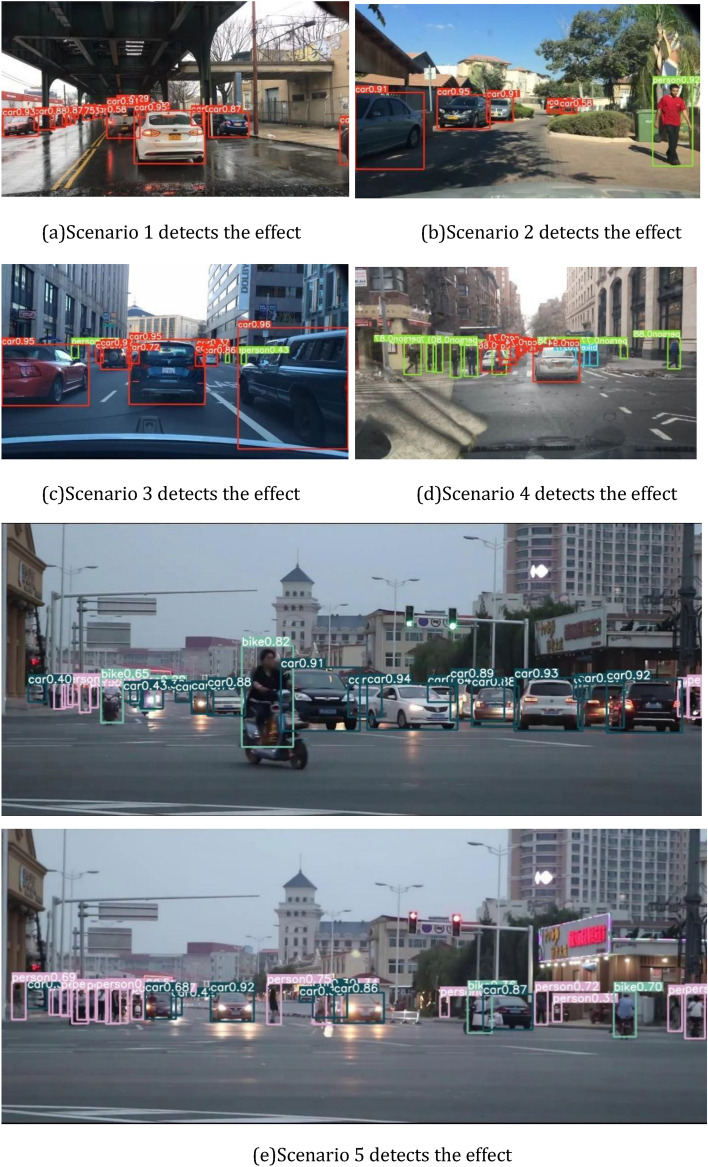
Detection performance of the proposed model.

After the introduction of the double head, the mAP has been greatly improved about 6%, indicating that the double head can improve the sensitivity of the network model to the classification task and regression task, and predict the classification information and regression information respectively without interfering with each other, improving the overall detection performance of the network. In order to improve the detection ability of small targets such as pedestrians and non-motor vehicles in road scenes, this paper adds a micro target detection layer to the original network structure. It can be seen from Tables 1 and 2 that on the two datasets, the mAP reaches 65.7% and 91.2% respectively. Adding the micro target detection layer can increase the concentration of the network on small objects, improve the proportion of the detection task for small objects, and improve detection accuracy. After introducing the ML-AFP, the mAP value of the final improved model reaches 66.8% and 93.2%, which can satisfy the detection performance in road scenes, and the detection effect is shown in Fig. 6.

### Comparison with other similar model

**Figure 7:**
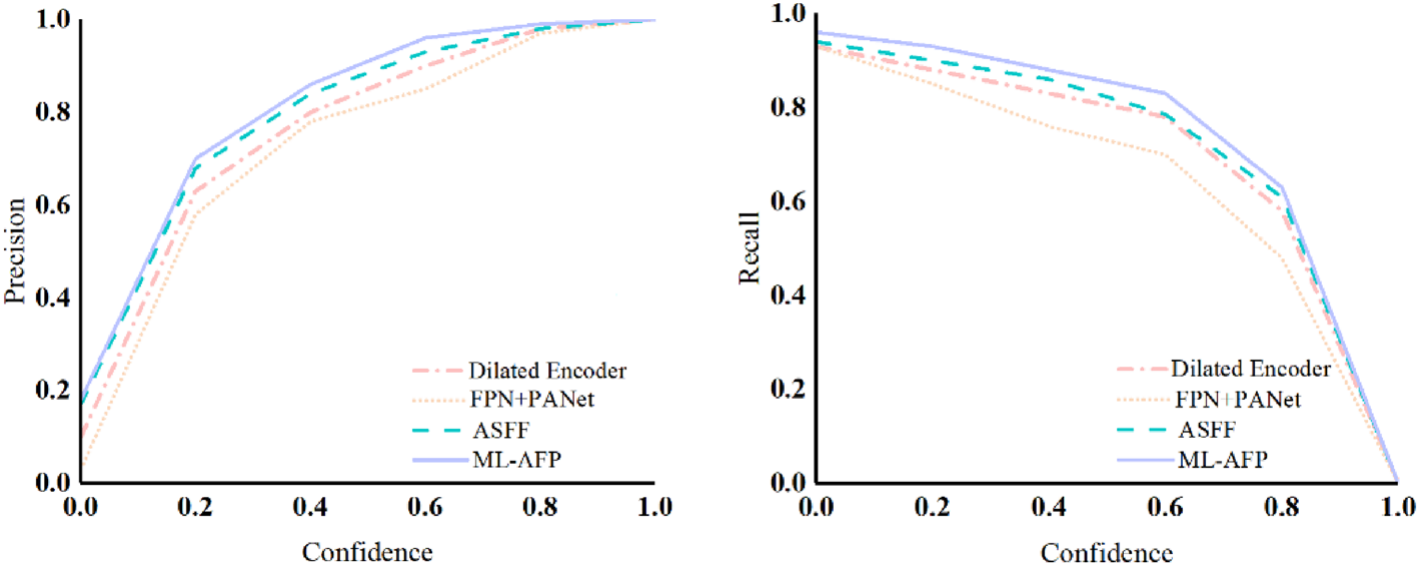
Comparison of precision and recall.

The feature pyramid is an important part of the YOLOv5 detection model. It is responsible for the multi-scale feature fusion and hierarchical detection of the detection model. The ML-AFP mechanism, adaptively spatial feature fusion (ASFF)^27^, and the Dilated Encoder structure in YOLOF^28^ were added to the YOLOv5 network structure, respectively. Compared with the FPN + PANet structure of YOLOv5 on KITTI dataset, The above structures are experimentally evaluated in three indicators of precision, recall, and mAP under different confidence levels. The precision comparison graph and recall comparison are shown in Fig. 7.

It can be seen from Fig. 7 that the precision and recall curves of the proposed ML-AFP mechanism are better than those of the comparison model under different confidence levels. Therefore, the effectiveness of the improved module can be verified by the image. Table 3 shows each categorie’s AP values and mAP of these structures under the detection of different target categories.

**Table 3:** Comparison of different structures in YOLOv5.

Model	mAP (%)	Person (%)	Bike (%)	Car (%)
YOLOv5 (FPN + PANet)	88.4	78.4	90.4	96.2
YOLOv5 (Dilated Encoder)	91.1	84.0	93.0	96.3
YOLOv5 (ASFF)	91.9	85.3	92.9	97.6
YOLOv5 (ML-AFP)	**92.1**	**85.5**	**93.1**	**97.7**

Significant values are in bold.

Table 3 shows that the mAP value of the Dilated Encoder is improved, which compared with FPN + PANet structure. The Dilated convolutions of different sizes in Dilated Encoder can adapt to different sizes of objects and improve the accuracy of different sizes of objects, and the residual structure can deepen the feature information. The structure can generate output features with multiple receptive fields, covering all the scales of objects. The mAP of ASFF structure is increased by 3.5 percentage points compared with FPN + PANet structure, and compared with Dilated Encoder, the mAP is increased by 0.8 percentage points. The 1 × 1 convolution and feature fusion method adopted by ASFF structure can better learn the contribution of different feature scales to the prediction feature map, and improve the prediction ability of the network. Compared with FPN + PANet, Dilated Encoder and ASFF, the ML-AFP mechanism proposed improves the MAP by 3.7%, 1.0%, and 0.2% respectively. The AP value of pedestrians is 7.1% higher than that of FPN + PANet, and the AP value of non-motor vehicles is 2.7% higher than that of FPN + PANet, The AP value of vehicles is increased by 1.5%, which is a good improvement. ML-AFP mechanism can better aggregate shallow spatial location information and deep high-level semantic information, and the pooling module in this mechanism can better distinguish the feature information of different levels, and differentiate the feature information of tiny, small, medium, and large object detection layers under the background of feature fusion. At the same time, for multi-class object detection tasks, the Sigmoid function can better integrate the learned weights into the spatial feature map and improve the performance of the FPN network.

## Conclusion

In this paper, we proposed an improved YOLOv5 multi object detection model for road scenes. A target detection layer and a double head mechanism are proposed to improve the detection accuracy of small objects such as pedestrians. For dense occlusion scenes on the road, Varifocal loss is used to improve the recall rate of the network model to solve the problem of target occlusion. The ML-AFP mechanism is proposed to enhance the feature extraction and feature fusion of the network to improve detection accuracy. Ablation and comparison experiments show that the proposed model has a great improvement in precision and recall rate compared with the original model and similar model. The model proposed in this paper considers the precision and recall rate which are the standards for detecting the quality of the model, however, there are other aspects should be focused on. In order to better meet the requirements of real-time detection, in future work, the forward inference speed and calculation amount of the model should be considered, as well as the adaptation of the model on different chips or platforms, should also be considered to meet the needs of industrial landing.

## Data Availability

The dataset collected and analysed during the current study is available from the corresponding author on request.
